# Is Aripiprazole an Effective Adjunct to Reduce Metabolic Adverse Effects Caused by Clozapine in Patients With Schizophrenia—A Systematic Review

**DOI:** 10.1002/npr2.70149

**Published:** 2026-07-28

**Authors:** Avtaar Singh, Soban Sadiq

**Affiliations:** ^1^ Kent and Medway Medical School University of Kent Canterbury UK

**Keywords:** antipsychotics, aripiprazole, clozapine, metabolic syndrome, schizophrenia

## Abstract

Clozapine is an atypical antipsychotic used in the treatment of schizophrenia. However, its use is associated with significant metabolic adverse effects, including hyperglycaemia, dyslipidaemia, and weight gain. Aripiprazole, a newer atypical antipsychotic with a different pharmacological profile, has been suggested to mitigate some of these metabolic side effects when used adjunctively. This systematic review assessed the evidence for the effectiveness of adjunctive Aripiprazole in reducing clozapine‐induced metabolic adverse effects. A systematic search was conducted across five academic databases, resulting in 52 articles. Following inclusion and exclusion criteria, eight studies were selected for narrative synthesis. These included randomized controlled trials, cohort studies, and case reports. The key metabolic outcomes assessed were glucose levels, lipid profiles, body weight, and waist circumference. Adjunctive Aripiprazole was associated with improvements in LDL and total cholesterol levels, as well as reductions in body weight in several studies. Fasting glucose levels and waist circumference showed limited or inconsistent changes. The overall evidence remains limited, particularly in terms of high‐quality, long‐term trials. This review suggests that Aripiprazole may offer some benefit in managing clozapine‐induced dyslipidaemia and weight gain. However, variability in outcomes, study design, and patient characteristics highlight the need for further research. Future studies should focus on larger, longer duration trials, broader patient demographics, and optimal dosing strategies to better evaluate the clinical utility of this combination therapy.

## Abstract 2

Adjunctive aripiprazole may provide modest improvements in lipid parameters and body weight in clozapine‐treated patients with schizophrenia, but evidence for glucose and waist circumference outcomes remains limited. Larger, long‐term randomized controlled trials are needed.

## Graphical abstract

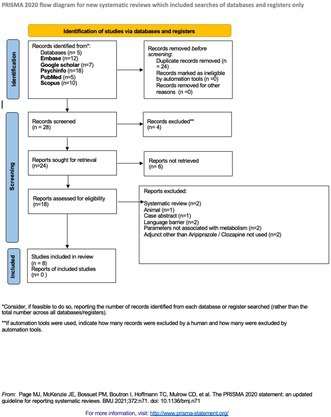

**Abbreviations**
5‐HT2: 5‐hydroxytryptamine. Letters or numbers after 5HT represent subtype of receptor
BDNF: brain derived neurotrophic factor
BMI: body mass index
CASP: critical appraisals skills programme
dL: decilitres
HbA1c: hemoglobin A1c
HDL: high‐density lipoprotein
kg: kilograms
LDL: low density lipoprotein
M3: muscarinic 3
mg: milligrams
PICO: population, intervention, control, and outcomes
RCT: randomized control trial
SGA: second generation antipsychotics

## 1 Introduction

Schizophrenia is a complex psychiatric disorder that is characterized by symptoms such as hallucinations, delusions, and lack of executive function [1]. Usually, the disease causes symptoms in early adulthood and is thought to be linked to increased dopaminergic activity in the mesolimbic neuronal pathway and a decrease in activity in the prefrontal cortical pathway [2]. Management of schizophrenia involves psychosocial as well as pharmacological treatment. Clozapine is an established antipsychotic prescribed for the treatment of schizophrenia where other pharmacological interventions have failed [3]. The mechanism of action of clozapine involves the blocking of 5‐HT2A/5‐HT2C serotonin receptors and the D1‐4 dopamine receptors, with the highest affinity for the D4 dopamine receptor [4].

Although treatment with clozapine has an established role in schizophrenia management, there are associated adverse metabolic effects that may pose challenges for both physicians and patients. Often these adverse effects lead to metabolic syndrome which includes, dyslipidaemia, increase glucose levels, and increase weight [5]. One area of research interest has been the use of Aripiprazole as an adjunct to clozapine. Aripiprazole is an atypical antipsychotic which is also used to treat schizophrenia and is unique in its mechanism of action (partial dopamine agonist) [6]. Given Aripiprazole has an agonist effect on both 5‐HT2C and 5‐HT1A receptors, it may have a role in mitigating the adverse metabolic effects caused by clozapine [7]. As clozapine has been proven to have many benefits on psychotic symptoms, if the adverse metabolic effects can be reduced, this could have a significant positive impact on the health and quality of life for individuals living with schizophrenia.

Although several studies have explored the use of adjunctive aripiprazole to mitigate clozapine‐associated metabolic adverse effects, the existing evidence is limited. In particular, uncertainty remains regarding the magnitude and consistency of effects across metabolic domains, as well as the quality of the available evidence. This systematic review was therefore undertaken to synthesize the existing literature and clarify the potential metabolic benefits of adjunctive aripiprazole in clozapine‐treated patients. The research question was derived using the PICO (population/problem, **i**ntervention, comparison, and outcome) framework (Appendix 1).

## 2 Methods

To develop a systematic review, a robust research method was applied. This involved, defining a research question, developing a search strategy, appropriate study selection, data extraction, and conducting a narrative synthesis. A thorough appropriate search string/terms were applied to the databases (Appendix 2). Although this was not a meta‐ analysis, ‘the preferred reporting Items for systematic reviews and meta‐analyses’ [8] (Appendix 3) was used to maintain an adequate standard and to ensure transparency [8]. It is important to highlight not all checklist items align with the meta‐analysis approach since this was a narrative synthesis. Ethics approval was not required as there were no active participants. Appendix 4 shows the NHS health authority research tool [9], and Appendix 5 shows the Kent and Medway REAG check list. In addition to the exclusion and inclusion criteria mentioned above, articles were also excluded due to duplication, unavailable text and papers not published in the English language.

Below is an outline of the methods used in this study using the PRISMA checklist.

### 2.1 Eligibility Criteria

#### 2.1.1 Population

Studies involving adult participants (≥ 18 years) diagnosed with schizophrenia or schizophrenia‐spectrum disorders, according to recognized diagnostic criteria (e.g., DSM or ICD), who were receiving clozapine treatment. No restriction was placed on gender or ethnicity.

#### 2.1.2 Intervention

Studies investigating adjunctive aripiprazole added to ongoing clozapine therapy, regardless of dose.

#### 2.1.3 Duration of Adjunctive Treatment

No minimum duration of adjunctive aripiprazole treatment was specified due to heterogeneity and the limited number of available studies.

#### 2.1.4 Outcomes

Studies were required to report at least one metabolic outcome, defined as follows:

- Body weight and/or body mass index (BMI)
- Lipid parameters, including total cholesterol, low‐density lipoprotein (LDL) cholesterol, high‐density lipoprotein (HDL) cholesterol, and/or triglycerides
- Glycaemic measures, including fasting plasma glucose, serum insulin, HbA1c, or indices of insulin resistance.
- Waist circumference, where available

#### 2.1.5 Study Design

Randomized controlled trials (RCTs) were prioritized; however, observational studies, retrospective cohort studies, and case reports were also included to maximize available evidence given the limited literature on this topic.

### 2.2 Information Sources

The databases selected were based on the research question and which databases would be most relevant. The databases selected were Embase (Ovid), Google Scholar, Psych info, PubMed, and Scopus.

### 2.3 Search Strategy Development Involved

- Defining a clear research question.
- Identifying key words.
- Identifying synonyms using Google search engine.
- Formulating a search string using key words/synonyms/Boolean operators/truncations.
- Search string was then applied to five academic search engines.
- The advance search option was used for each five of the search engines.
- The search was limited to title and abstract only.

### 2.4 Selection Process

- Initial review of the article title and abstract.
- Review of study design, patient demographics, intervention, outcomes measured.
- Determining the clarity of the research question.
- Reviewing the presentation of results—tables, figures, and statistical analysis performed.
- Reviewing the discussion, interpretation of results, and observing if limitations are acknowledged.
- Analyzing the overall organization of the paper.
- Observing any risk of bias.
- Ethics approval and disclosure of conflicts of interest.
- Peer review status.
- Journal impact factor.
- Screening was initially conducted by one reviewer and subsequently reviewed by a second reviewer to reduce the risk of selection bias.

### 2.5 Data Collection Process

Data extraction was performed independently by two reviewers and recorded in a predefined Excel spreadsheet for data tabulation (Appendix 6). Data items were determined a priori based on the research question, existing literature, and predefined inclusion and exclusion criteria. Extracted variables included study design, study duration, sample size, participant characteristics, baseline metabolic measurements, intervention details, and reported outcomes. Any disagreements identified during the review process were resolved through discussion until consensus was reached.

### 2.6 Data Items

All outcomes sought were related to metabolic parameters that clozapine has been shown to affect: Glucose levels, lipid profile, weight gain and waist circumference. Additional variables extracted included study design, duration, sample size, participant characteristics, baseline metabolic status, and intervention characteristics. Where data were missing or unclear in the original reports, no assumptions or imputations were made, and such data were recorded as not reported.

### 2.7 Study Risk of Bias Assessment

Bias assessment and overall analysis within the selected studies was performed using the Cochrane Risk of bias tool [10] (Appendix 7) and the CASP tool (Appendix 8) [11] for the RCTs; the Modified Downs and Black tool [12] (Appendices 9 and 10) for the open label single arm studies. For the case reports and the retrospective study, the JBL tool [13] (Appendix 11) was used. The Cochrane risk of bias tool is a more detailed assessment of bias compared to the CASP tool; therefore, both tools were used for the RCTs.

### 2.8 Synthesis Methods

Initially the data was inserted into a table and reviewed for the most appropriate method of synthesis. Key outcomes were assessed and compared and contrasted across all studies using the core text. Heterogeneity was assessed by visual inspection of the individual papers and considered study design, patient demographic, sample size, intervention, variation in statistical analysis, and outcomes measured. Given the heterogeneity, a narrative synthesis was performed using the extracted data from the tables (Appendix 6).

### 2.9 Reporting Bias

To minimize any bias due to missing results in the synthesis, a comprehensive search strategy was formed using the PICO criteria with a clear outline of the inclusion and exclusion criteria to ensure a broad range of results was produced from multiple search engines. Formulation of the individual included study characteristics was performed and presented in Appendix (12). Consistency and discrepancies of the results were examined and discussed in the results section.

Certainty assessment: carried out by assessing each paper for study design, sample size, appropriateness, evaluating the risk of bias, assessing for patterns across the studies and the overall coherence and quality of the study. This was also contained within the CASP tool, modified Downs and Black tool, and JBL. Certainty of evidence was assessed narratively, considering study design, risk of bias, consistency of findings, and precision, with overall certainty judged as low to moderate.

## 3 Results

**FIGURE 1:**
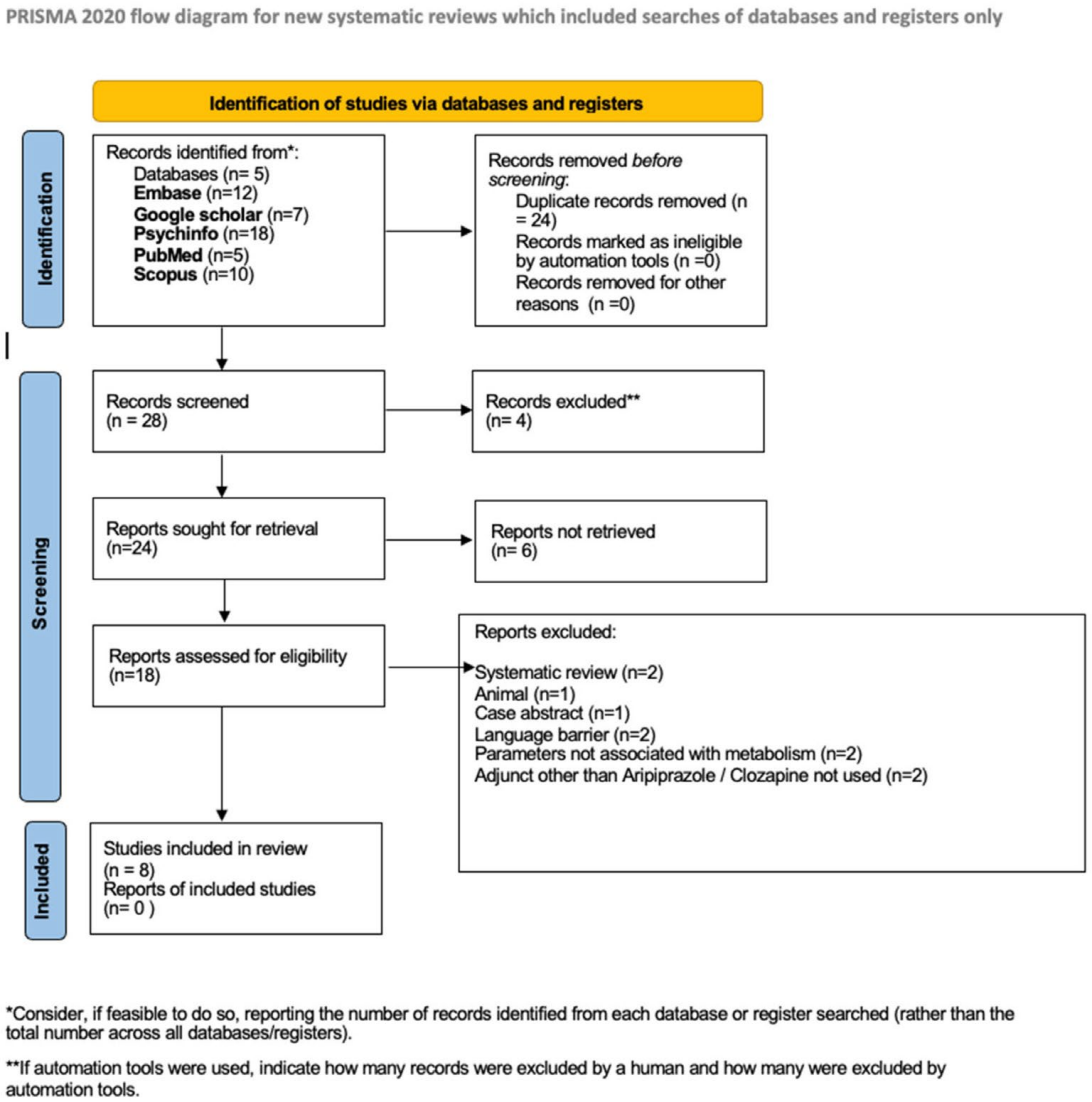
PRISMA flow diagram of study identification, screening, eligibility, and inclusion (Adapted from Page et al. [14]).

The PRISMA flow diagram (Figure 1) shows that the search retrieved a total of 52 results (Embase:12, Google Scholar: 7, Psychinfo: 18, PubMed: 5, Scopus: 10). Of these 52 results, studies were excluded for the following reasons: duplicate results (24), irrelevant based on title/abstract (4), record not retrieved (6), systematic review (2), case abstract (1), language barrier (2), outcomes not related to metabolic parameters (2), adjunct other than Aripiprazole used (2), and finally 1 animal study. The final number of studies included in the review was 8. Three of these studies were double‐blind RCTs, two open label studies, two case reports, and one retrospective cohort study. The justification for using the selected papers and their respective details are shown below (Appendix 12).

The reason for including different study designs was due to the limited number of available literature based on the inclusion and exclusion criteria. The details of the study characteristics (as mentioned above) are shown in Appendix 6. Key outcomes were extracted from all of the eight papers and analyzed thoroughly. The key outcomes in this analysis are glucose levels, lipid profile, body weight, and waist circumference. Appendix 13 displays more detail of the results from the individual papers. Given the heterogeneity of study designs and outcome measures, results are presented by study design, with RCTs presented first as the highest level of evidence, followed by non‐randomized studies and case reports, which are interpreted as supportive or hypothesis‐generating evidence. A narrative summary of the results is shown below (Table 1). RCTs represent the highest level of evidence and therefore form the primary basis for the core conclusions of this review.

**TABLE 1:** Summary of results.

Outcome	Direction of effect	Evidence base	Statistical significance	Certainty (GRADE)
Lipid profile (total cholesterol, LDL)	Improvement (reduction)	Randomized controlled trials with supportive non‐randomized evidence	Statistically significant reductions reported in several RCTs	Low–moderate
Body weight/BMI	Modest reduction	RCTs and non‐randomized studies	Mixed findings; more consistent in longer duration studies	Low–moderate
Glucose parameters (fasting glucose, HbA1c, insulin)	No consistent change	Primarily RCT evidence	Largely nonsignificant across trials	Low
Waist circumference	Inconsistent	Limited RCT evidence	Significant reduction reported mainly in longer duration trials	Low

### 3.1 Evidence From Randomized Control Trials

Three double‐blind RCTs evaluated the metabolic effects of adjunctive aripiprazole in patients receiving clozapine treatment [7, 15, 16]. Effect sizes, baseline and follow‐up values, and measures of uncertainty are reported where available from the original studies; where such data were not explicitly reported, this is indicated to maintain transparency. (Table 2).

**TABLE 2:** Summary of metabolic outcomes and effect estimates from randomized controlled trials.

Study	Design	Attrition	Duration	Aripiprazole dose	Outcome	Aripiprazole versus placebo (change from baseline)	Statistical result
Fan et al. [7]	RCT	21.1% (8/38)	8 weeks	15 mg	LDL‐C	−15.1 versus +4.4 mg/dL	*p* = 0.019
					Weight	−1.5 versus +0.3 kg	NS (*p* = 0.109)
					Glucose	+0.9 versus +7.5 mg/dL	NS (*p* = 0.206)
Fleischhacker et al. [16]	RCT	Reported 15%–20%	16 weeks	5–15 mg	Weight	−2.53 versus −0.38 kg	*p* < 0.001
					LDL‐C	−10.3% versus 0.0%	*p* = 0.003
					Waist circumference	−2.0 versus 0 cm	*p* = 0.001
					Glucose	+0.6% versus +1.2%	0.828
Chang et al. [15]	RCT	9.7% (6/62)	8 weeks	5–30 mg	Triglycerides	−31.1 versus +24.4 mg/dL	*p* < 0.01
					Weight	−1.2 versus −0.6 kg	NS (*p* = 0.36)
					Glucose	−10.2 versus +3.4 mg/dL	NS (*p* = 0.56)
					LDL‐C	−8.7 versus −3.9 mg/dL	NS (*p* = 0.22)

*Note:* Values represent mean change from baseline.

Abbreviation: NS, not statistically significant.

#### 3.1.1 Glucose Levels

Across RCTs, adjunctive aripiprazole did not demonstrate consistent or statistically significant improvements in glucose‐related outcomes. Measures including fasting glucose, serum insulin, and HbA1c largely remained unchanged over study durations ranging from 8 to 16 weeks [7, 15, 16].

#### 3.1.2 Lipid Profile

RCT evidence showed more favorable effects on lipid parameters. Reductions in total cholesterol and LDL cholesterol were reported in trials evaluating adjunctive aripiprazole compared with placebo, whereas changes in triglycerides and HDL cholesterol were less consistent [7, 16]. One RCT did not demonstrate statistically significant lipid changes [15].

#### 3.1.3 Bodyweight and Body Mass Index

Weight and BMI outcomes varied across RCTs. The longest duration trial (16 weeks) reported a statistically significant reduction in body weight in the aripiprazole group [16], whereas shorter‐duration trials showed smaller or non‐significant changes [7, 15].

#### 3.1.4 Waist Circumference

One RCT reported a statistically significant reduction in waist circumference following adjunctive aripiprazole [7], while other trials reported no meaningful change over the study period [15, 16].

#### 3.1.5 Dose Response and Individual Subject Factors

The RCT studies included in this review implemented varying dosages of Aripiprazole and involved subjects with somewhat diverse characteristics such as baseline health status and age. The studies had little focus on specific dose responses and lacked subgroups based on subject characteristics; therefore, there was not adequate information to draw conclusions regarding this.

### 3.2 Evidence From Non‐RCTs

Non‐randomized evidence included two open‐label trials and one retrospective cohort study [17, 18, 19].

Across these studies, adjunctive aripiprazole was generally associated with reductions in body weight and improvements in lipid parameters, particularly total and LDL cholesterol [17, 18, 19]. One retrospective cohort study reported a statistically significant reduction in fasting glucose levels following aripiprazole augmentation [17]; however, this finding was not consistently replicated in RCTs.

Interpretation of these findings is limited by the absence of control groups, small sample sizes, and the potential influence of confounding factors such as concomitant medications, lifestyle changes, and variations in clozapine dosing.

### 3.3 Evidence From Case Reports

Two case‐based studies reported metabolic improvements following long‐term aripiprazole augmentation in patients receiving clozapine [20, 21]. Reported outcomes included reductions in body weight, lipid levels, and waist circumference, with one case reporting a marked decrease in waist circumference over a 20‐month follow‐up period [21].

While these findings are clinically interesting, they represent low‐level evidence and lack control groups and formal statistical testing. As such, they should be interpreted as hypothesis‐generating rather than confirmatory.

### 3.4 Safety and Psychiatric Outcomes

Across the included studies, psychiatric outcomes, adverse events, discontinuation rates, and clozapine dose changes were variably reported. RCTs generally required participants to be on a stable clozapine dose prior to and during adjunctive treatment, whereas non‐randomized and retrospective studies did not consistently report clozapine dose stability. (Table 3).

**TABLE 3:** Psychiatric outcomes, adverse events, and treatment discontinuation across included studies.

Study (design; duration)	Psychiatric outcomes (scales/results)	Safety endpoints (AEs incl. akathisia/EPS)	Discontinuations/attrition	Clozapine dose changes/confounding note
Fleischhacker et al. [16] (RCT; 16 weeks double‐blind)	PANSS total: no significant between‐group difference; CGI‐Improvement and Investigator's Assessment Questionnaire favored aripiprazole (abstract)	Most common AEs (≥ 5%): nausea, anxiety, insomnia, etc.; EPS‐related AEs reported; akathisia (3/109)	NR in extracted table text (trial reports treatment‐emergent AEs; overall tolerability described as comparable)	Stable clozapine dose required; no clozapine dose‐reduction strategy described—metabolic change unlikely explained by planned dose reduction
Fan et al. [7] (RCT; 8 weeks)	PANSS referenced in methods; psychiatric efficacy results not clearly reported in extracted text (metabolic primary)	AEs (≥ 5% and ≥ 2× placebo): overarousal, drowsiness, itching, headache, chest pain, etc.	30 completed (16 aripiprazole; 14 placebo) (abstract). Reasons for attrition NR in extracted text	Entry required stable clozapine dose; no dose‐reduction strategy described
Chang et al. [15] (RCT; 8 weeks)	Negative symptoms improved vs. placebo (BPRS negative symptom subscale; SANS) from Week 4 onward; most other efficacy measures NS	No serious AEs; UKU mean change NS overall. Aripiprazole associated with decreased sleep duration and orthostatic hypotension; DIEPSS change NS	NR in extracted text	NR regarding clozapine dose changes in extracted text; interpret metabolic changes assuming dose broadly stable unless stated otherwise
Henderson et al. [19] (open‐label; 6 weeks)	Trial examined positive/negative symptoms; specific scale results NR in extracted snippet	NR in extracted snippet	10 enrolled; 8 completed 6 weeks; 2 ended at Week 4	Dose‐reduction confounding can't be excluded unless paper states clozapine dose remained stable
De Risio et al. [17] (retrospective cohort)	Clinical outcome in clozapine‐resistant schizophrenia reported (details NR in extracted text)	AEs/side effect reporting limited/NR in extracted text	NR.	Retrospective routine‐care setting: dose adjustments could confound metabolic changes unless explicitly ruled out
Gupta et al. [18] (open‐label; 12 weeks)	Psychiatric outcomes NR in extracted text	AEs/discontinuations NR in extracted text (check full paper tables if available)	NR in extracted text	Open‐label adjunct design: if clozapine/other antipsychotic doses changed, this may confound metabolic outcomes
Karunakaran et al. [20] (case series)	Symptom outcomes NR in extracted text	AEs/EPS reporting NR in extracted text	NR	Case series in routine practice: clozapine dose changes are plausible confounders unless explicitly reported as stable
Masopust et al. [21] (case report; 20 months)	Psychiatric outcomes NR in extracted text	AEs/EPS NR in extracted text	NR	Single‐patient design; attribution depends heavily on whether clozapine dose remained stable (NR here)

Abbreviation: NR, not reported.

## 4 Discussion

### 4.1 Summary of Key Findings

This systematic review examined four metabolic outcomes associated with adjunctive aripiprazole in patients receiving clozapine: glucose parameters, lipid profile, body weight/BMI, and waist circumference. Across RCTs, there was no consistent evidence to support a clinically meaningful effect of adjunctive aripiprazole on fasting glucose, HbA1c, or other glycaemic measures.

In contrast, low‐to‐moderate certainty evidence from RCTs suggests modest improvements in lipid parameters, particularly reductions in total cholesterol and LDL cholesterol. Changes in triglycerides and HDL cholesterol were less consistent.

Body weight and BMI outcomes were variable, with modest reductions observed in some studies, particularly those of longer duration, while others demonstrated no statistically significant change. Waist circumference did not show consistent improvement across studies, with meaningful reductions reported primarily in longer duration trials (other than the case report mentioned above).

Using GRADE principles to assess the certainty of evidence derived primarily from RCTs, the certainty of evidence varied by metabolic outcome. For lipid parameters, there is low‐to‐moderate certainty evidence, downgraded due to short study duration and modest sample sizes despite generally consistent findings. For body weight and BMI, there is low‐to‐moderate certainty evidence, downgraded due to heterogeneity in effect size and study duration. For glucose‐related outcomes (including fasting glucose, serum insulin, and HbA1c), the certainty of evidence is low, reflecting inconsistent findings and limited follow‐up. For waist circumference, the certainty of evidence is also low due to few studies reporting this outcome and inconsistent effects.

### 4.2 Interpretation of Findings

The most consistent metabolic finding across higher quality evidence was a modest reduction in lipid parameters, particularly total and LDL cholesterol, associated with adjunctive aripiprazole in clozapine‐treated patients. This effect was observed primarily in RCTs and was supported by non‐randomized studies, which should be interpreted as supplementary evidence. Given the elevated cardiovascular risk associated with long‐term clozapine treatment, these findings may be clinically relevant; however, lipid modification represents only one component of overall metabolic risk management.

Evidence supporting beneficial effects on body weight and BMI was less consistent and appeared influenced by study duration, baseline metabolic status, and potential confounders such as lifestyle interventions, concomitant medications, and clozapine dose. Although some studies suggested modest weight reduction, these findings should be interpreted cautiously, and adjunctive aripiprazole should not be viewed as a substitute for established non‐pharmacological interventions such as diet and exercise.

Adjunctive aripiprazole did not demonstrate a consistent benefit on waist circumference, which may reflect the need for longer follow‐up durations to detect changes in central adiposity. The lack of consistent improvement in both waist circumference and glucose parameters suggests that adjunctive aripiprazole may have a limited impact on insulin resistance, particularly in the short term.

The inconsistent findings observed for glucose‐related outcomes and waist circumference across studies may be attributable to several factors. First, study duration was relatively short in most RCTs (8–16 weeks), which may be insufficient to detect changes in glucose metabolism or central adiposity, outcomes that often evolve more slowly than lipid parameters or body weight. Second, baseline metabolic status varied across study populations, with some participants exhibiting preexisting metabolic abnormalities and others remaining metabolically stable at baseline, potentially limiting the capacity to detect change.

Overall, low‐to‐moderate certainty evidence suggests that adjunctive aripiprazole may confer modest lipid‐lowering benefits, with less consistent effects on weight and minimal evidence of benefit for glucose metabolism. These findings should be interpreted within the context of short study durations and methodological heterogeneity.

### 4.3 Limitations

Several limitations should be considered when interpreting these findings.

First, study durations varied substantially, with most RCTs limited to short‐term follow‐up (8–16 weeks). As metabolic changes, particularly central adiposity and insulin resistance, may require longer durations to manifest, the available evidence may underestimate longer term effects.

Second, sample sizes varied widely across studies, ranging from single case reports to larger RCTs. Smaller studies were inherently underpowered to detect modest metabolic changes, limiting the generalizability of their findings.

Third, participant demographics were relatively homogeneous, with a predominance of middle‐aged, male, Caucasian participants. This limits extrapolation of findings to younger patients, females, and ethnically diverse populations. In addition, clozapine dose and duration prior to study entry varied considerably and may have influenced baseline metabolic risk and treatment response.

Fourth, heterogeneity in study design, outcome definitions, and reporting precluded quantitative meta‐analysis. While risk‐of‐bias assessments suggested relatively low risk in RCTs, non‐randomized studies and case reports were subject to inherent limitations, including lack of control groups and potential selection and observer bias.

Fifth, although measures were taken to improve methodological rigor and reduce bias, some risk of selection or interpretation bias may remain.

Sixth, across the included studies, psychiatric symptom outcomes, adverse events, discontinuation rates, and clozapine dose changes were variably reported. Overall, adjunctive aripiprazole was not associated with worsening of psychotic symptoms in RCTs, although increased rates of activation‐related adverse events, particularly akathisia, were reported in some studies. Discontinuation rates were generally low and comparable between treatment groups where reported. RCTs typically required participants to be on a stable clozapine dose prior to and during adjunctive treatment, whereas non‐randomized and retrospective studies did not consistently report clozapine dose stability (see Table 3).

Finally, publication bias should be considered when interpreting the findings of this review. Formal assessment of publication bias (e.g., funnel plot analysis) was not feasible due to the small number of included studies and the heterogeneity of study designs. Smaller studies and case reports reporting favorable metabolic effects of adjunctive aripiprazole may be more likely to be published, whereas studies demonstrating null or negative findings may be underrepresented. This may result in an overestimation of the metabolic benefits observed in the narrative synthesis.

## 5 Conclusion and Summary

This systematic review conducted a structured, evidence‐based assessment of whether adjunctive aripiprazole is associated with improvements in metabolic adverse effects among patients with schizophrenia receiving clozapine. Specifically, the review evaluated the effects of adjunctive aripiprazole on glucose parameters, lipid profile, body weight/BMI, and waist circumference, synthesizing evidence from RCTs, non‐randomized studies, and case reports. The inclusion of diverse study designs reflects the limited and heterogeneous evidence base currently available for this clinical question.

Overall, low‐to‐moderate certainty evidence from RCTs suggests that adjunctive aripiprazole may be associated with modest improvements in lipid parameters, particularly reductions in total and LDL cholesterol. Evidence for effects on body weight and BMI was inconsistent, with modest reductions observed in some studies, particularly those of longer duration, while others demonstrated no statistically significant change. In contrast, there was no consistent evidence to support clinically meaningful improvements in glucose parameters or waist circumference with adjunctive aripiprazole.

Taken together, these findings suggest that adjunctive aripiprazole may offer limited metabolic benefit, primarily in relation to dyslipidaemia, in patients receiving clozapine. However, the magnitude of observed effects was modest, and findings were influenced by short study durations, variability in baseline metabolic status, and methodological heterogeneity. As such, adjunctive aripiprazole should not be viewed as a comprehensive strategy for managing clozapine‐associated metabolic risk.

In conclusion, current evidence does not support broad metabolic benefit of adjunctive aripiprazole in clozapine‐treated patients but suggests a potential role in improving lipid parameters for selected individuals. However, the magnitude of these effects appears modest and should be interpreted within the context of short study durations and methodological heterogeneity. Adjunctive aripiprazole should therefore not be considered a primary strategy for managing clozapine‐associated metabolic risk but may represent a potential adjunctive option in specific clinical contexts. Further well‐powered, longer duration RCTs are required to clarify the durability of these effects, evaluate safety and psychiatric outcomes, and better define the clinical role of this combination therapy.

## Author Contributions

**Avtaar Singh** conceived and designed the study, developed the search strategy, conducted the literature search, performed the analysis, and drafted the manuscript. **Soban Sadiq** contributed to study screening review, participated in data extraction and review, contributed to critical revisions of the manuscript, and provided recommendations for methodological and structural improvements.

## Funding

The authors have nothing to report.

## Conflicts of Interest

The authors declare no conflicts of interest.

## Data Availability

No new datasets were generated or analyzed during the current study. All data used in this systematic review were obtained from previously published studies cited within the manuscript.

## Appendix 1 Framework (PICO)

**Table:**

	PICO
Population	Individuals with a diagnosis of schizophrenia/psychotic disorders treated with clozapine
Intervention	Aripiprazole as an adjunct
Comparison	Treated with clozapine only or clozapine and placebo
Outcomes	Change in metabolic parameters
Time	Within the last 15 years
Research question	Is aripiprazole an effective adjunct to reduce metabolic adverse effects caused by clozapine in schizophrenic patients
Hypothesis	H_0_ Aripiprazole is an effective adjunct therapy to reduce the metabolic effects caused by clozapine

## Appendix 2 Data Bases and Search String

**Table:**

Database (platform)	Search date	Exact search string used	Limits/filters applied	Hits
Embase (Ovid)	March 2024	(‘clozapine’/exp. OR clozapine) AND (‘aripiprazole’/exp. OR aripiprazole) AND (adjunct OR augment* OR add* OR combin*) AND (metabol* OR weight OR bmi OR glucose OR lipid* OR ldl OR triglyceride* OR diabet* OR insulin)	Human; English language	12
Google Scholar	March 2024	(“clozapine” AND “aripiprazole” AND (metabolic OR weight OR BMI OR glucose OR lipid OR LDL OR triglyceride OR diabetes OR insulin))	First 200 results screened (sorted by relevance)	7
PsycINFO	March 2024	(clozapine AND aripiprazole AND (adjunct OR augment* OR add* OR combin*) AND (metabol* OR weight OR BMI OR glucose OR lipid* OR LDL OR triglyceride* OR diabet* OR insulin))	Human; English language	18
PubMed	March 2024	(clozapine[Title/Abstract]) AND (aripiprazole[Title/Abstract]) AND (adjunct OR augment* OR add* OR combin*) AND (metabol* OR weight OR BMI OR glucose OR lipid* OR LDL OR triglyceride* OR diabet* OR insulin)	Humans; English language	5
Scopus	March 2024	TITLE‐ABS‐KEY (clozapine AND aripiprazole AND (adjunct OR augment* OR add* OR combin*) AND (metabol* OR weight OR BMI OR glucose OR lipid* OR LDL OR triglyceride* OR diabet* OR insulin))	—	10

## Appendix 3 PRISMA Check List (Page et al. [14])

**Table:**

Section and topic	Item #	Checklist item	Location where item is reported
**Title**			
Title	1	Identify the report as a systematic review	Title
**Abstract**			
Abstract	2	See the PRISMA 2020 [5] for Abstracts checklist	Abstract
**Introduction**			
Rationale	3	Describe the rationale for the review in the context of existing knowledge	Introduction
Objectives	4	Provide an explicit statement of the objective(s) or question(s) the review addresses.	Introduction
**Methods**			
Eligibility criteria	5	Specify the inclusion and exclusion criteria for the review and how studies were grouped for the syntheses	Methods point 1
Information sources	6	Specify all databases, registers, websites, organizations, reference lists and other sources searched or consulted to identify studies. Specify the date when each source was last searched or consulted	Appendix 2
Search strategy	7	Present the full search strategies for all databases, registers and websites, including any filters and limits used	Appendix 2
Selection process	8	Specify the methods used to decide whether a study met the inclusion criteria of the review, including how many reviewers screened each record and each report retrieved, whether they worked independently, and if applicable, details of automation tools used in the process	Methods‐ selection process
Data collection process	9	Specify the methods used to collect data from reports, including how many reviewers collected data from each report, whether they worked independently, any processes for obtaining or confirming data from study investigators, and if applicable, details of automation tools used in the process	Methods‐ data collection
Data items	10a	List and define all outcomes for which data were sought. Specify whether all results that were compatible with each outcome domain in each study were sought (e.g., for all measures, time points, analyzes), and if not, the methods used to decide which results to collect	Methods data items
	10b	List and define all other variables for which data were sought (e.g., participant and intervention characteristics, funding sources). Describe any assumptions made about any missing or unclear information	Methods data items
Study risk of bias assessment	11	Specify the methods used to assess risk of bias in the included studies, including details of the tool(s) used, how many reviewers assessed each study and whether they worked independently, and if applicable, details of automation tools used in the process	Methods – Risk of bias
Effect measures	12	Specify for each outcome the effect measure(s) (e.g., risk ratio, mean difference) used in the synthesis or presentation of results	Results
Synthesis methods	13a	Describe the processes used to decide which studies were eligible for each synthesis (e.g., tabulating the study intervention characteristics and comparing against the planned groups for each synthesis (item #5))	Methods – data synthesis results
	13b	Describe any methods required to prepare the data for presentation or synthesis, such as handling of missing summary statistics, or data conversions	Methods‐ data collection
	13c	Describe any methods used to tabulate or visually display results of individual studies and syntheses	Results
	13d	Describe any methods used to synthesize results and provide a rationale for the choice(s). If meta‐analysis was performed, describe the model(s), method(s) to identify the presence and extent of statistical heterogeneity, and software package(s) used	Methods – data synthesis
	13e	Describe any methods used to explore possible causes of heterogeneity among study results (e.g., subgroup analysis, meta‐regression)	Discussion – narratively
	13f	Describe any sensitivity analyzes conducted to assess robustness of the synthesized results	Not performed
Reporting bias assessment	14	Describe any methods used to assess risk of bias due to missing results in a synthesis (arising from reporting biases)	Risk of bias tools from Appendix 7 to 11
Certainty assessment	15	Describe any methods used to assess certainty (or confidence) in the body of evidence for an outcome	Discussion – interpretation of findings
**Results**			
Study selection	16a	Describe the results of the search and selection process, from the number of records identified in the search to the number of studies included in the review, ideally using a flow diagram	Results
	16b	Cite studies that might appear to meet the inclusion criteria, but which were excluded, and explain why they were excluded	Results
Study characteristics	17	Cite each included study and present its characteristics	Appendix 6
Risk of bias in studies	18	Present assessments of risk of bias for each included study	Appendices 7, 8, 9 and 10 and discussion
Results of individual studies	19	For all outcomes, present, for each study: (a) summary statistics for each group (where appropriate) and (b) an effect estimate and its precision (e.g., confidence/credible interval), ideally using structured tables or plots	Results
Results of syntheses	20a	For each synthesis, briefly summarize the characteristics and risk of bias among contributing studies	Results, Appendix 6, discussion—limitations, Appendices 7, 8, 9, and 10
	20b	Present results of all statistical syntheses conducted. If meta‐analysis was done, present for each the summary estimate and its precision (e.g., confidence/credible interval) and measures of statistical heterogeneity. If comparing groups, describe the direction of the effect	Results – narrative
	20c	Present results of all investigations of possible causes of heterogeneity among study results	Not done
	20d	Present results of all sensitivity analyzes conducted to assess the robustness of the synthesized results	Not formally assessed – discussed in limitations
Reporting biases	21	Present assessments of risk of bias due to missing results (arising from reporting biases) for each synthesis assessed	Discussion – narratively
Certainty of evidence	22	Present assessments of certainty (or confidence) in the body of evidence for each outcome assessed	
**Discussion**			
Discussion	23a	Provide a general interpretation of the results in the context of other evidence	Discussion—interpretation
	23b	Discuss any limitations of the evidence included in the review	Discussion—limitations
	23c	Discuss any limitations of the review processes used	Discussion—limitations
	23d	Discuss implications of the results for practice, policy, and future research	Discussion—limitations
**Other information**			
Registration and protocol	24a	Provide registration information for the review, including register name and registration number, or state that the review was not registered	Not registered
	24b	Indicate where the review protocol can be accessed, or state that a protocol was not prepared	No formal protocol
	24c	Describe and explain any amendments to information provided at registration or in the protocol	N/A
Support	25	Describe sources of financial or nonfinancial support for the review, and the role of the funders or sponsors in the review	No funding
Competing interests	26	Declare any competing interests of review authors	Title page
Availability of data, code and other materials	27	Report which of the following are publicly available and where they can be found: template data collection forms; data extracted from included studies; data used for all analyzes; analytic code; any other materials used in the review	All data extracted and analyzed in this review were derived from published studies and are presented within the manuscript. No analytical code was generated and no additional materials are publicly available

## Appendix 6 Study Characteristics

**Table:**

	Study type	Subjects recruited	Subjects completed	Inclusion criteria	Primary outcomes	Statistical analysis methods	Study duration	Patient demographic	Aripiprazole dose (mg/day)	Limitations
Fan et al. [7]	RCT‐ double blind	44	30	18–65 years; diagnosis of schizophrenia or schizoaffective disorder; treatment with clozapine for at least 1 year; stable dose of clozapine for at least 1 month	Metabolic parameters	Performed using SAS (version 9.2, SAS Institute, Cary, NC, USA) *t*‐test/Fishers, chi square, ANCOVA, Cohens *d*	8 weeks	**Aripiprazole group**: Mean age: 44.3 Gender: 13 (male), 3 (female) Race:11 Caucasian, 4 African American, 1 other Diabetic *n* = 4 Diagnosis, 13 (schizophrenia), 3 (schizoaffective disorder) Mean dose clozapine (mg/day) 397 **Placebo group** Mean age: 44.2 Gender: 9 (male), 5 (female) Race: 13 Caucasian, 10 African American, 1 other Diabetic *n* = 5 Diagnosis, 7 (schizophrenia), 7 (schizoaffective disorder) Mean dose clozapine (mg/day) 400 **All patients had treatment with clozapine for at least 1 year prior to entry and a stable dose for 1 month	15	Small sample size Study duration
Fleischhacker et al. [16]	RCT‐ double blind	230	190 double blind phase Open label extension 167	18–65 with diagnosis of schizophrenia according to DSM IV TR, on stable dose of clozapine for 3 months (200–900 mg/day)	Mean change from baseline body weight	SAS statistical soft‐ ware, version 8.2 or higher ANCOVA LOCF	16 weeks 12 weeks open label extension	**Aripiprazole:** Mean age: 37.6 Gender: 68 (male), 40 (female) Race: 103 white, 5 other Diagnosis, All (schizophrenia), Duration of clozapine treatment before trial 64.1 months Mean Dose of clozapine before trial: 383.8 mg/dL **Placebo:** Mean age: 40.5 Gender: 66 (male), 33 (female) Race: 94 white, 5 other Diagnosis, All (schizophrenia), different subtypes Duration of clozapine treatment before trial 63.3 months Mean dose of clozapine before trial: 362.6 mg/dL	5–15	Subject demographics lacks variability (predominantly white participants)
Chang et al. [15]	RCT‐double blind	62	52	18–65 with diagnosis of schizophrenia on stable dos of clozapine for 8 weeks, treated for at least 1 year	Change in BPRS score	Chi square Fishers	8 weeks	**Aripiprazole:** Mean age: 33.2 Gender: 22 (male), 7 (female) Diagnosis, All (schizophrenia), Mean duration of clozapine before trial entry‐ 750 days Mean dose of clozapine 304.3 mg **Placebo:** Mean age: 31.7 Gender: 26 (male), 6 (female) Diagnosis, All (schizophrenia), Mean duration of clozapine before trial entry‐ 744.1 days Mean dose of clozapine 290.6 mg	5–30	Primary outcome measure was not metabolic

## Appendix 7 Cochrane Risk of Bias Tool (Y = yes, PY = probably yes, *N* = no, PN = probably not)

**Table:**

Signaling questions	Fan et al. [7]	Fleischhacker et al. [16]	Chang et al. [15]	Response options
**Domain 1: Risk of bias arising from the randomization process**				
1.1. Was the allocation sequence random?	Y	Y	Y	Y/PY/PN/N/NI
1.2. Was the allocation sequence concealed until participants were enrolled and assigned to interventions?	PY	PY	PY	Y/PY/PN/N/NI
1.3. Did baseline differences between intervention groups suggest a problem with the randomization process?	N	N	N	Y/PY/PN/N/NI
Risk‐of‐bias judgment	Low	Low	Low	Low/High/Some concerns
Optional: What is the predicted direction of bias arising from the randomization process?				NA/Favors experimental/Favors comparator/Towards null/Away from null/Unpredictable
**Domain 2: Risk of bias due to deviations from the intended interventions (effect of assignment to intervention)**				
2.1. Were participants aware of their assigned intervention during the trial?	N	N	N	Y/PY/PN/N/NI
2.2. Were carers and people delivering the interventions aware of participants' assigned intervention during the trial?	PN	PN	PN	Y/PY/PN/N/NI
2.3. *If Y/PY/NI to 2.1 or 2.2*: Were there deviations from the intended intervention that arose because of the trial context?				NA/Y/PY/PN/N/NI
2.4. *If Y/PY to 2.3*: Were these deviations likely to have affected the outcome?				NA/Y/PY/PN/N/NI
2.5. *If Y/PY/NI to 2.4*: Were these deviations from intended intervention balanced between groups?				NA/Y/PY/PN/N/NI
2.6. Was an appropriate analysis used to estimate the effect of assignment to intervention?	Y	Y	Y	Y/PY/PN/N/NI
2.7. *If N/PN/NI to 2.6*: Was there potential for a substantial impact (on the result) of the failure to analyze participants in the group to which they were randomized?				NA/Y/PY/PN/N/NI
Risk‐of‐bias judgment	Low	Low	Low	Low/High/Some concerns
**Domain 3: Missing outcome data**				
3.1. Were data for this outcome available for all, or nearly all, participants randomized?	Y	Y	Y	Y/PY/PN/N/NI
3.2. *If N/PN/NI to 3.1*: Is there evidence that the result was not biased by missing outcome data?				NA/Y/PY/PN/N
3.3. *If N/PN to 3.2*: Could missingness in the outcome depend on its true value?				NA/Y/PY/PN/N/NI
3.4. *If Y/PY/NI to 3.3*: Is it likely that missingness in the outcome depended on its true value?				NA/Y/PY/PN/N/NI
Risk‐of‐bias judgment	Low	Low	Low	Low/High/Some concerns
Optional: What is the predicted direction of bias due to missing outcome data?				NA/Favors experimental/Favors comparator/Towards null/Away from null/Unpredictable
**Domain 4: Risk of bias in measurement of the outcome**				
4.1. Was the method of measuring the outcome inappropriate?	Y	Y	Y	Y/PY/PN/N/NI
4.2. Could measurement or ascertainment of the outcome have differed between intervention groups?	PN	PN	PN	Y/PY/PN/N/NI
4.3. *If N/PN/NI to 4.1 and 4.2*: Were outcome assessors aware of the intervention received by study participants?				NA/Y/PY/PN/N/NI
4.4. *If Y/PY/NI to 4.3*: Could assessment of the outcome have been influenced by knowledge of intervention received?				NA/Y/PY/PN/N/NI
4.5. *If Y/PY/NI to 4.4*: Is it likely that assessment of the outcome was influenced by knowledge of intervention received?				NA/Y/PY/PN/N/NI
Risk‐of‐bias judgment	Low	Low	Low	Low/High/Some concerns
Optional: What is the predicted direction of bias in measurement of the outcome?				NA/Favors experimental/Favors comparator/Towards null/Away from null/Unpredictable
**Domain 5: Risk of bias in selection of the reported result**				
5.1. Were the data that produced this result analyzed in accordance with a prespecified analysis plan that was finalized before unblinded outcome data were available for analysis?	NI	NI	NI	Y/PY/PN/N/NI
Is the numerical result being assessed likely to have been selected, on the basis of the results, from…				
5.2. … multiple eligible outcome measurements (e.g., scales, definitions, time points) within the outcome domain?	N	N	N	Y/PY/PN/N/NI
5.3. … multiple eligible analyzes of the data?	N	N	N	Y/PY/PN/N/NI
Risk‐of‐bias judgment	Low	Low	low	Low/High/Some concerns

## Appendix 8 CASP Tool

**Table:**

CASP tool	Effects of adjunctive treatment with aripiprazole on body weight and clinical efficacy in schizophrenia patients treated with clozapine: A randomized, double‐blind, placebo‐controlled trial [16]	Metabolic effects of adjunctive aripiprazole in clozapine‐treated patients with schizophrenia [7]	Aripiprazole augmentation in clozapine‐treated patients with refractory schizophrenia: an 8‐week, randomized, double‐blind, placebo‐controlled trial [15]
Did the review address a clearly focused question?	Yes Focused population sample to those with a diagnosis of schizophrenia taking clozapine Focused intervention with either Aripiprazole or Placebo Focused comparison to placebo group Focused measured outcome – Body weight	Yes Focused population sample to those with a diagnosis of schizophrenia taking clozapine Focused intervention with either Aripiprazole or Placebo Focused comparison to placebo group Focused measured outcome: Glucose metabolism, lipid levels, BMI	Yes Focused population sample to those with a diagnosis of schizophrenia taking clozapine Focused intervention with either aripiprazole or placebo Focused comparison to placebo group Focused measured outcome – (1) Symptoms (2) Prolactin (3) Triglycerides
Was the assignment of participants to interventions randomized?	Yes: Achieved through computer generated randomization. Helpful control of bias.	Yes: However, it was not stated how randomization was achieved.	Yes Used random numbers
Were all the participants who entered the study accounted for at conclusion?	Yes: Clear table showing participants entered and those discontinued	Yes: Clear figure showing participants entered and those discontinued	Yes: Clearly stated number of participants/dropouts and completion
(1) Were the participants blind to the intervention they were given (2) Were the investigators blind to the intervention they were giving participants (3) Were the people assessing/analyzing outcomes blind?	(1) Yes (2) Yes (3) Can't tell	Not able to tell, not clearly stated.	(1) Yes (2) Yes (3) Yes
Were the study groups similar at the randomized control trial?	Largely similar demographics although some slight discrepancies which are to be expected. Weight & Age was similar between the groups.	The two groups differed in race at a trend level (*p* = 0.084); There were no significant differences between the two groups in age, gender, education level, diagnosis (schizophrenia or schizoaffective disorder), tobacco use, diabetes status, and the daily clozapine dosage (*p* values > 0.1) (Table 1).	Considerably more male than female Age 18–51 No comment on ethnic group
Apart from the experimental intervention, did each study group receive the same level of care?	This is not clearly stated however: Clozapine dose in both groups remained constant, however Aripiprazole dose was variable. Intervals of assessment were the same for each group	This is not clearly stated. In addition, clozapine dose was not stated, however: Aripiprazole dose remained constant among both groups Intervals of assessment were the same for each group	Can't tell
Were the effects of intervention reported comprehensively?	Yes: Clear statistics with the use of figures Power was calculated Primary outcome measure was mean change in body weight. Secondary outcomes were change from base line glucose, LDL, triglycerides, total cholesterol & HDL. Body weight % change was reported at each stated interval; however, the other secondary outcomes were measured at the last week comparing them to baseline. Clinical efficacy was also reported ANCOVA statical test was used compare man change in body weight *p* values were reported	Group comparisons were performed using the independent *t*‐test for continuous variables, and the Fisher's exact test or chi‐square test for categorical variables. Analysis of covariance (ANCOVA) was used to compare change scores from baseline to week 8 between the two groups controlling for baseline scores. For all analyzes, a *p* value < 0.05 (two‐tailed) was used for statistical significance. For those continuous variables with findings at a trend level, Cohen's *d* effect sizes were calculated. Outcomes measured: Glucose clearance, lipid levels, body weight, BMI, waist circumference. *p* values reported	Yes: Clear statistics with use of figures Compared baseline starting weight/bmi and lipid profile
Was the precision of the estimate of intervention or treatment effect reported?	Mean change in body weight was presented using a 95% confidence interval	No confidence intervals stated	No confidence intervals stated
Do the benefits of the experimental intervention outweigh the harms and cost?	Cost analysis was not reported. Adverse effects were reported, and these have to be considered. Nausea was the most common reported adverse effects	Cost analysis was not reported. Adverse effects were reported, and these have to be considered. Nasal congestion was the most common adverse effect	Cost analysis was not reported Adverse effects not clearly stated
Can the results be applied to your local population/context?	Yes	Larger sample size would be required	Larger sample size would be required
Would the experimental intervention provide greater value to the people in your care than any of the existing interventions?	At the time this paper was published, further exploration of this question is warranted following the results. There are practical considerations to implementing this treatment, such as cost and adverse reactions and clinical efficacy of treatment.	At the time this paper was published, further exploration of this question is warranted following the results. There are practical considerations to implementing this treatment, such as cost and adverse reactions and clinical efficacy of treatment.	Yes Clear statistical change in metabolic profile (positive)
Is it a relevant journal?	Yes International Journal of Neuropsychopharmacology Peer reviewed	Yes Acta Psychiatrica Scandinavica Peer reviewed Journal	Yes

## Appendix 9 Modified Downs & Black Tool [19]

**Table:**

Checklist item	Assessment
Is the hypothesis/aim/objective of the study clearly described?	Yes: to study the effect of adjunct aripiprazole on weight, lipid, and glucose metabolism, as well as positive and negative symptoms of schizophrenia
Are the main outcomes clearly described?	Yes: primary outcomes were weight, lipid and glucose metabolism, plus positive and negative symptoms
Are participant characteristics clearly described?	No: Reported in the text but not easy to locate
Are the interventions clearly described?	Yes: 5 mg aripiprazole
Are principal confounders described?	No
Are the main findings clearly described?	Yes: Clear tables
Random variability reported?	Standard deviations and *p* values reported; no confidence intervals
Adverse events reported?	Yes
Loss to follow‐up described?	Reason for dropout reported
Participants representative?	Small sample; difficult to determine, but demographics typical of clozapine‐treated population
Settings representative?	Yes: Mental health community center
Statistical tests appropriate?	Two‐tailed *t*‐tests
Compliance reliable?	Not stated how compliance was measured
Outcome measures valid/reliable?	Yes: Overall methodological approach, intervention and results clearly described

## Appendix 10 Modified Downs & Black Tool [18]

**Table:**

Checklist item	Assessment
Is the hypothesis/aim/objective of the study clearly described?	Yes: To study the effect of adjunct low‐dose aripiprazole on weight and metabolic parameters
Are the main outcomes clearly described?	Yes: Weight change primary; metabolic parameters secondary
Are participant characteristics clearly described?	Yes: Clear table shown
Are the interventions clearly described?	Yes: Low‐dose aripiprazole
Are principal confounders described?	Weight‐loss medications excluded
Are the main findings clearly described?	Yes: Clear tables
Random variability reported?	Confidence intervals and standard deviations reported
Adverse events reported?	Yes
Loss to follow‐up described?	Reason for dropout reported
Participants representative?	Small sample; difficult to determine
Settings representative?	Yes: Institute of Mental Health, Singapore
Statistical tests appropriate?	SPSS LOCF used
Compliance reliable?	Medication compliance assessed by pill counts
Outcome measures valid/reliable?	Yes: Pill count

## Appendix 11 JBL Bias Tool

**Table:**

	Yes	No	Unclear	N/A
Karunakaran et al. [20]				
Were patient's demographic characteristics clearly described?			✔	
Was the patient's history clearly described and presented as a timeline?	Yes, clear history presented in text ✔			
Was the current clinical condition of the patient on presentation clearly described?	Yes: information on current diagnosis ✔			
Were diagnostic tests or assessment methods and the results clearly described?	Yes, clear methodology explaining how the cases were reviewed ✔			
Was the intervention(s) or treatment procedure(s) clearly described?	Yes ✔			
Was the post‐intervention clinical condition clearly described?				N/A ✔
Were adverse events (harms) or unanticipated events identified and described?	✔			
Does the case report provide takeaway lessons?	✔			
Masopust et al. [21]				
Were patient's demographic characteristics clearly described?	✔			
Was the patient's history clearly described and presented as a timeline?	✔			
Was the current clinical condition of the patient on presentation clearly described?	✔			
Were diagnostic tests or assessment methods and the results clearly described?	✔			
Was the intervention(s) or treatment procedure(s) clearly described?	✔			
Was the post‐intervention clinical condition clearly described?				✔
Were adverse events (harms) or unanticipated events identified and described?		✔		
Does the case report provide takeaway lessons?	✔			
De Risio et al. [17]				
Were there clear criteria for inclusion in the case series?			Not clearly stated but inferred from text. ✔	
Was the condition measured in a standard, reliable way for all participants included in the case series?				N/A ✔
Were valid methods used for identification of the condition for all participants included in the case series?	Yes: All patients were admitted to psychiatric unit and required treatment ✔			
Did the case series have consecutive inclusion of participants?			Not clear ✔	
Was there clear reporting of the demographics of the participants in the study?	Yes: Age, gender, baseline condition. Other characteristics unclear i.e., ethnicity, socioeconomic statis ✔			
Was there clear reporting of clinical information of the participants?	Yes. Information on psychiatric condition and baseline metabolic measurements ✔			
Were the outcomes or follow up results of cases clearly reported?	Yes: Clear table showing results ✔			
Was there clear reporting of the presenting site(s)/clinic(s) demographic information?	Yes, location of clinic/site presented ✔			
Was statistical analysis appropriate?	Yes: Paired *t*‐tests ✔			

## Appendix 12 Selected Study Details and Justification

**Table:**

#	Citation	Link/DOI	Reason for choice
1	Fan, X., Borba, C., Copeland, P., Hayden, D., Freudenreich, O., Goff, D.C., and Henderson, D.C. (2012) ‘Metabolic effects of adjunctive aripiprazole in clozapine‐treated patients with schizophrenia’, *Acta Psychiatrica Scandinavica*, 127(3), pp. 217–226	doi: 10.1111/acps.12009	Randomized double‐blind controlled trial; 8 weeks; various metabolic parameters measured; specific to the review question
2	Fleischhacker, W.W., Heikkinen, E.M., Olie, J.P., Landsberg, W., Dewaele, P., McQuade, R.D., Loze, J.Y., Hennicken, D., and Kerselaers, W. (2010) ‘Effects of adjunctive treatment with aripiprazole on body weight and clinical efficacy in schizophrenia patients treated with clozapine: A randomized, double‐blind, placebo‐controlled trial’, *The International Journal of Neuropsychopharmacology*, 13(08), pp. 1115–1125	doi: 10.1017/S1461145710000490	Randomized double‐blind controlled trial; 16 weeks; large sample size; highly cited; clear reporting; specific to the review question
3	Chang, J.S., Ahn, Y.M., Park, H.J., Lee, Y.L., Kim, S.H., and Kang, U.G. (2008) ‘Aripiprazole augmentation in clozapine‐treated patients with refractory schizophrenia’, *The Journal of Clinical Psychiatry*, 69(5), pp. 720–731	doi: 10.4088/JCP.v69n0505	Randomized double‐blind controlled trial; 8 weeks; clear statistics; specific to the review question
4	Henderson, D.C., Kunkel, L., Nguyen, D.D., Borba, C.P., Daley, T.B., Louie, P.M., Freudenreich, O., Cather, C., Evins, A.E. and Goff, D.C. (2006). An exploratory open‐label trial of aripiprazole as an adjuvant to clozapine therapy in chronic schizophrenia. *Acta Psychiatrica Scandinavica*, 113(2), pp.142–147. Available at: doi: https://doi.org/10.1111/j.1600‐0447.2005.00612.x	doi: 10.1097/01.pra.0000358312.99233.ef	Clear aims and objectives; single‐arm design; substantial study length (12 weeks); participants matched inclusion criteria; clear statistics; credible authors
5	Gupta, B., Chee, K.S., Neo, L.Q., Tang, C., Hariram, J., Tan, G.C., Basu, S., Appan, D.P., Ting, C.C., Abdim, E., and Lee, J. (2021) ‘Effect of aripiprazole as an adjunct to atypical antipsychotics on weight and metabolic profile: A 12‐week open‐label trial’, *Therapeutic Advances in Psychopharmacolog*y, 11, p. 204512532110467.	doi: 10.1177/20451253211046765	Open‐label trial (12 weeks); clear strengths and weaknesses; credible authors; clear data; relevant outcomes
6	Karunakaran, K., Tungaraza, T.E. and Harborne, G.C. (2006) ‘Is clozapine—aripiprazole combination a useful regime in the management of treatment‐resistant schizophrenia?’, *Journal of Psychopharmacology*, 21(4), pp. 453–456	doi: 10.1177/0269881106068289	Case report series; simple design; multiple case reports; long average exposure duration (as reported in the paper).
7	Masopust, J., Tůma, I. and Libiger, J. (2008) ‘Adjunctive aripiprazole decreased metabolic side effects of clozapine treatment’, *Neuro Endocrinology Letters*, 29(4), pp. 435–437	PubMed: https://pubmed.ncbi.nlm.nih.gov/18766167/	Case study; long follow‐up (20 months); one of few studies including a younger patient; relevant metabolic outcomes
8	De Risio, A., Pancheri, A., Simonetti, G., Giannarelli, D., Stefanutto, L., and Gentile, B. (2011) ‘Add‐on of aripiprazole improves outcome in clozapine‐resistant schizophrenia’, *Progress in Neuro‐Psychopharmacology and Biological Psychiatry*, 35(4), pp. 1112–1116	doi: 10.1016/j.pnpbp.2011.03.011	Retrospective cohort study; multiple outcomes measured including symptom control; clear statistics; provides historical perspective

## Appendix 13 Key Findings/Results From Studies

**Table:**

	Glucose levels & insulin changes	Lipid profile	Body weight/BMI	Waist circumference	Adverse reactions
Fan et al. [7]	**Statistically significant findings:** 8‐week change – improvement in glucose clearance (*p* = 0.010). Method: Baseline venous blood sample Glucose tolerance test (venous) Not clear at what intervals measurements were taken after baseline **Insignificant/no change:** Plasma glucose, HbA1C, acute insulin response, disposition index	**Statistically significant findings:** 8‐week reduction in plasma LDL levels & LDL particle number Aripiprazole group: Direct LDL Mean baseline 101.0 mg/dL Direct LDL Mean 8 week change −15.1 mg/dL LDL particle number (nmol/L) 1610 LDL particle number (nmol/L) mean change −376 *p* = 0.019 Placebo: Direct LDL Mean baseline 101.6 mg/dL Direct LDL Mean 8 week change 22.5 mg/dL LDL particle number (nmol/L) 1628 LDL particle number (nmol/L) mean 8 week change −36 *p* = 0.035 **Insignificant/no change:** Plasma total cholesterol, HDL, triglycerides, LDL particle size, HDL particle number concentration and VLDL particle number concentration Method: Laboratory assay Not clear at what intervals measurements were taken after baseline	**Statistically significant findings:** N/A **Insignificant/no change:** No statistically significant difference between placebo and treatment group *p* = > 0.1 Not clear at what intervals measurements were taken after baseline	**Statistically significant findings:** N/A **Insignificant/no change:** No statistically significant difference between placebo and treatment group *p* = > 0.1 Not clear at what intervals measurements were taken after baseline	Overarousal 3 Drowsiness 4 Itching 2 Headache 3 Chest pain 3 Back pain 2 Nasal congestion 5 Hypersalivation 3 Nausea 4 Vomiting 3 Stomach discomfort
Fleischhacker et al. [16]	**Statistically significant findings**: N/A **Insignificant/no change:** No statistically significant difference in fasting glucose (*p* = 0.282) 87 were in the placebo group and 96 in the Aripiprazole group. Percentage change 1.2 ± 2.0 and 0.7 ± 1.9 respectively with *p* = 0.282 Method: not clear	**Statistically significant findings:** Decrease in total cholesterol (*p* = 0.002) and LDL cholesterol (0.003) from baseline compared to placebo. Aripiprazole: Total cholesterol mean baseline 211.8 ± 4.3 Total cholesterol % change −6.9 ± 1.2 LDL cholesterol mean baseline 122.7 ± 3.8 LDL cholesterol % change −10.3 ± 2.2 Placebo: Total cholesterol mean baseline 202.6 ± 4.6 Total cholesterol % change −1.2 ± 1.4 LDL cholesterol mean baseline 116.2 ± 4.0 LDL cholesterol % change 0.00 ± 2.6 **Insignificant/no change:** HDL, triglycerides Method: not clear	**Statistically significant findings**: Patients on adjunctive aripiprazole experienced a significantly greater mean decrease in body weight compared to those on adjunctive placebo (2.53 kg vs. 0.38 kg, respectively), with a mean treatment difference of 2.15 kg. This difference was statistically significant from week 6 through to the endpoint. From week 2 reductions in BMI greater in the Aripiprazole group (*p* = 0.001) Aripiprazole group Baseline weight (kg) 94.7 ± 18.2 Baseline BMI (kg/m^2^) 31.4 ± 6.4 Placebo group Baseline weight (kg) 89.8 ± 16.4 Baseline BMI (kg/m^2^) 30.1 ± 5 Method: measured at baseline and then at Week 1, 4, 6, 8, 12	**Statistically significant findings**: Patients on adjunctive Aripiprazole showed mean reduction of 2 cm. (*p* = 0.001) Method: not clear	Nausea, Diarrhea, Vomiting, Anxiety, Insomnia, Headache, Nasopharyngitis, Cough EPS‐related (total) 4 (4.1) Akathisia
Chang et al. [15]	**Statistically significant findings**: N/A **Insignificant/no change:** There were no significant differences between the aripiprazole and placebo groups in terms of fasting and 2‐h postprandial blood sugar levels, no comment on insulin Method: not clear, assume capillary blood close	**Statistically significant findings**: N/A **Insignificant/no change:** No statistically significant changes were observed in the levels of total cholesterol HDL (high‐density lipoprotein), or LDL (low‐density lipoprotein) cholesterol between the two groups from baseline to the study endpoint Method: not clear but serum is mentioned	**Statistically significant findings**: N/A **Insignificant/no change:** No statistical difference in bodyweight or BMI observed Method: weight measured at baseline and then at 1, 2, 4, and 8 weeks	**Statistically significant findings**: N/A **Insignificant/no change:** No statistical difference in waist circumference Method: measured at baseline and then at 1,2,4, and 8 weeks	Gastrointestinal discomfort, anxiety, palpitations, in the treatment group
Henderson et al. [19]	**Statistically significant findings**: N/A **Insignificant findings/no change** Fasting glucose and insulin concentrations did not decrease significantly Investigated fasting glucose levels (*t* = −95, *p* = 0.39), insignificant change. mean Base line glucose 121.8 ± 69.2 mg/dL, mean end point glucose 130.2 ± 69.2 Insulin HOM‐IR decreased from 10.76 to 4.59 (*t* = 1.35, *p* = 0.24), insignificant change Method: blood sample assays At baseline then at 4 and 6 weeks	**Statistically significant findings**: Significant decreases were observed in total serum cholesterol (*p* = 0.002), total triglycerides (0.04), and HDL cholesterol (*p* = 0.02). Total serum cholesterol mean baseline (mg/dL) 211.0 ± 26.9 Total serum cholesterol mean end point 184.4 ± 26.9 Total triglycerides mean baseline (mg/dL) 274.2 ± 228.7 Total triglycerides mean end point 176.1 ± 106 No figures provided for HDL apart from *p* value as stated above **Insignificant findings/no change** LDL cholesterol also decreased, though not significantly. Method: blood sample assays At baseline then at 4 and 6 weeks	**Statistically significant findings**: Significant decreases were observed in weight *p* = 0.003) and BMI (*p* = 0.004), with both measures reduced in nine out of 10 subjects Mean baseline weight (kg) 99.8 Mean endpoint weight (kg) 97.1 Mean baseline BMI (kg/m^2^) 33.1 Mean endpoint BMI (kg/m^2^) 32.1 **Insignificant findings/no change** N/A Method: blood sample assays At baseline then at 4 and 6 weeks	**Statistically significant findings**: N/A **Insignificant findings/no change:** No statistically significant change Method: blood sample assays At baseline then at 4 and 6 weeks	Not reported
Gupta et al. [18]	**Statistically significant findings** N/A **Insignificant findings** No statistical difference in fasting glucose levels *N* = 55 Investigated fasting glucose Baseline average fasting glucose 4.89 mmol/L, after 12 weeks, average 4.84, *p* = 0.491, insignificant change Method: venous blood sample at baseline and then at 12 weeks) end point. Serum assay using Roche diagnostics C702 and C513, HbA1C	**Statistically significant findings** Total cholesterol (mmol/L) Mean baseline—4.82 Mean at 12 weeks‐ 4.35 < 0.0001 LDL cholesterol (mmol/L) Mean at baseline—2.57 Mean at 12 weeks‐ 1.19 *p* = 0.044 Triglycerides (mmol/L) Mean at baseline—2.95 Mean at 12 weeks‐ 2.37 *p* = 0.038 HDL cholesterol (mmol/L) Mean at baseline 1.11 Mean at 12 weeks 2.29 *p* = 0.016 **Insignifcant findings** Methods: Method: venous blood sample at baseline and then at 12 weeks) end point. Serum assay using Roche diagnostics C702 and C513, HbA1C	**Statistically significant findings** For clozapine users: 92.9% (13 out of 14) recorded weight loss. *p* = 0.002 Mean baseline weight (kg) 84.22 Mean endpoint weight (kg) 82.16 **Insignificant findings** N/A Method: baseline measurement then at 0, 4, 8, and 12 weeks)	**Statistically significant findings** N/A No statistically significant change Method: baseline measurement then at 0, 4, 8, and 12 weeks)	Anxiety, dizziness, sedation, nausea, increased appetite, restlessness, dystonia, somnolence, dry mouth, insomnia, depression, tremor, headache (severe), blurred vision, vomiting, constipation, salivary hypersecretion, fatigue, rash, shortness of breath, reduced appetite, urinary frequency, confusion
Karunakaran et al. [20]	**Statistically significant findings** N/A **Insignificant findings:** No statistically significant difference in fasting glucose levels (*p* = 0.499) Insulin not reported *N* = 24 Investigating fasting glucose Mean baseline fasting glucose 6.1, after augmentation of Aripiprazole mean 5.8 mmo/L, *p* = 0.499, insignificant change Method: not clear as multiple cases reported	**Statistically significant findings** N/A **Insignificant findings:** Mean HDL increased from 1.09 mmol/L to 1.17 mmol/L (*p* = 0.064), insignificant Serum total cholesterol reduced but insignificant From 5.32 to 4.85 (*p* = 0.168) Method: not clear as multiple cases reported	**Statistically significant findings** 75% of patients lost weight (average 5.1 kg, 5.4% of pre‐augmentation weight). (*p* = 0.002) Pre‐augmentation mean weight (kg) 95.3 Post‐augmentation mean weight (kg) 90.2 **Insignificant findings:** N/A Method: not clear as multiple cases reported	Not reported	One patient developed severe dyskinesia affecting the trunk and limbs and the other was suspected to have developed atypical neuroleptic malignant syndrome. In both cases, the observed side effect abated when aripiprazole was discontinued
Masopust et al. [21]	Not reported	Reduction in total cholesterol by 2.5 in 20 months. Baseline on clozapine for 8 months 6.98 (mmol/L) Reduction in triglycerides of 6.15 in 20 months Baseline on clozapine 6.88 No statistical tests Method: not reported, but measured before clozapine commenced, then 8 months after start of clozapine, then measured when patient receiving clozapine and Aripiprazole after 2/4/8 and 20 months	Patient lost 20.8 kg in 20 months No statistical analysis Baseline weight on clozapine for 8 months 107.8 kg Method: not reported, but measured before clozapine commenced, then 8 months after start of clozapine, then measured when patient receiving clozapine and aripiprazole after 2/4/8 and 20 months	Reduced by 21 cm in 20 months No statistical analysis Baseline waist circumference after 8 months clozapine 115 cm Method: not reported, but measured before clozapine commenced, then 8 months after start of clozapine, then measured when patient receiving clozapine and aripiprazole after 2/4/8 and 20 months. at 20 months waist circumference measured as 94 cm	Not reported
De Risio et al. [17]	**Statistically significant findings** Reduction in fasting glucose levels Investigated fasting glucose Baseline blood glucose average 102.25. End point blood glucose average 88.500 *p* = 0.000, statically significant change Methods: review so individual methods not reported **Insignificant findings** N/A	**Statistically significant findings** Total cholesterol levels demonstrated a reduction from baseline to endpoint. *p* = 0.00 Baseline total cholesterol mean (mg/dL) 244.31 Endpoint total cholesterol 196.43 HDL cholesterol, however, showed an increase during the same period. *p* = 0.00 Baseline HDL cholesterol 32.87. End point HDL cholesterol 38.7 Methods: review so individual methods not reported **Insignificant findings** N/A	**Statistically significant findings** Reduction in weight and bmi stated but no clear figures Methods: review so individual meth odds not reported **Insignificant findings** N/A	Not reported	Not reported
